# Development of Effective Connectivity during Own- and Other-Race Face Processing: A Granger Causality Analysis

**DOI:** 10.3389/fnhum.2016.00474

**Published:** 2016-09-22

**Authors:** Guifei Zhou, Jiangang Liu, Xiao Pan Ding, Genyue Fu, Kang Lee

**Affiliations:** ^1^School of Computer and Information Technology, Beijing Jiaotong UniversityBeijing, China; ^2^Dr. Eric Jackman Institute of Child Study, University of TorontoToronto, ON, Canada; ^3^Department of Psychology, National University of SingaporeSingapore, Singapore; ^4^Department of Psychology, Hangzhou Normal UniversityHangzhou, China; ^5^Department of Psychology, Zhejiang Normal UniversityJinhua, China

**Keywords:** other-race effect, developmental ORE, face recognition, fNIRS, effective connectivity, Granger causal network

## Abstract

Numerous developmental studies have suggested that other-race effect (ORE) in face recognition emerges as early as in infancy and develops steadily throughout childhood. However, there is very limited research on the neural mechanisms underlying this developmental ORE. The present study used Granger causality analysis (GCA) to examine the development of children's cortical networks in processing own- and other-race faces. Children were between 3 and 13 years. An old-new paradigm was used to assess their own- and other-race face recognition with ETG-4000 (Hitachi Medical Co., Japan) acquiring functional near infrared spectroscopy (fNIRS) data. After preprocessing, for each participant and under each face condition, we obtained the causal map by calculating the weights of causal relations between the time courses of [oxy-Hb] of each pair of channels using GCA. To investigate further the differential causal connectivity for own-race faces and other-race faces at the group level, a repeated measure analysis of variance (ANOVA) was performed on the GCA weights for each pair of channels with the face race task (own-race face vs. other-race face) as the within-subject variable and the age as a between-subject factor (continuous variable). We found an age-related increase in functional connectivity, paralleling a similar age-related improvement in behavioral face processing ability. More importantly, we found that the significant differences in neural functional connectivity between the recognition of own-race faces and that of other-race faces were modulated by age. Thus, like the behavioral ORE, the neural ORE emerges early and undergoes a protracted developmental course.

## Introduction

From early childhood, children typically have asymmetrical exposures to own- and other-race faces. They see own-race faces significantly more frequently than other-race faces (Rennels and Davis, 2008; Sugden et al., 2014). One of the consequences of this early asymmetrical face exposure is that we recognize own-race faces faster and more accurately than other-race faces. This phenomenon is referred to as the other-race effect (ORE; for reviews, see Meissner and Brigham, 2001; Sporer, 2001; Anzures et al., 2013a). ORE has been replicated in adults from different racial backgrounds using various paradigms (Malpass and Kravitz, 1969; Levin, 2000; Ge et al., 2009; Liu et al., 2015). ORE has been also consistently observed in childhood (for a review, see Anzures et al., 2013a). Numerous developmental studies have revealed that ORE emerges as early as in infancy (Sangrigoli and de Schonen, 2004; Hayden et al., 2007; Kelly et al., 2009) and develops steadily throughout childhood (Chance et al., 1982; Pezdek et al., 2003; Walker and Hewstone, 2006; Goodman et al., 2007; Anzures et al., 2014).

In addition to the behavioral ORE, recent functional neuroimaging studies have found that adults show differential neural responses when recognizing own-race vs. other-race faces (Golby et al., 2001; Ito and Urland, 2005; Kim et al., 2006; Feng et al., 2011; Natu et al., 2011; Senholzi and Ito, 2013; Liu et al., 2015; for a review, see: Ito and Bartholow, 2009). This difference in neural response to different racial faces is referred to as the Neural Other-Race Effect (NORE, Ding et al., 2014). For example, using event related potential (ERP), several studies (Ito and Urland, 2005; Natu et al., 2011; Senholzi and Ito, 2013) found that own-race faces and other-race faces elicit significant differences in the N170, an important ERP component related to face processing (Bentin et al., 1996). Using the functional magnetic resonance imaging (fMRI) methodology, Golby et al. (2001) and Kim et al. (2006) found greater neural responses for own-race faces than for other-race faces in the right fusiform face area (FFA, Kanwisher et al., 1997). Further, using fMRI and the dynamic causal modeling, Liu et al. (2015) found that recognizing own- vs. other-race faces engenders differential causal functional connectivity between the key cortical regions specialized in face processing.

In contrast to the extensive studies of the behavioral ORE in children and adults, and those of the neural ORE in adults, little is known about the neural correlates of own- vs. other-race face processing in children. To date, no studies have examined the origins of neural ORE and how it develops with age, with two exceptions. One study by Balas et al. (2011) used the ERP technique. It demonstrated a neural ORE whereby 9-month-old infants respond differentially to own-race faces and other-race faces, consistently with the behavioral findings with infants (Anzures et al., 2013a). Another study by Ding et al. (2014) used functional near-infrared spectroscopy (fNIRS) to investigate the neural correlates of own- and other-race face recognition in children between 7 and 13 years of age. They found that regardless of age, neural activations in the right MFG/IFG, and the left cuneus were greater when recognizing other-race faces than own-race faces. Further, they found that different age groups presented different correlational patterns between the neural ORE in these regions and those in other brain regions. For example, with increased age, the numbers of the significant correlations between the occipital ORE areas and the frontal areas increased. Ding et al. (2014) speculated that functional neural network underlying the neural ORE might undergo developmental changes whereby the posterior-frontal integration of the key cortical regions involved in face processing differs for own- vs. other race face. However, Ding et al. (2014) only examined the correlations of fNIRS activation levels between different brain regions rather than the relations of dynamic responses of these regions. Thus, they could not provide direct evidence supporting their speculation.

The present study aimed to bridge this significant gap in the literature. More specifically, we used Granger causality analysis (GCA, Granger, 1969) to examine the developmental patterns of neural networks underlying the ORE in children between 3 and 13 years of age. The most distinctive advantage of GCA over the traditional Pearson correlational analysis is that the GCA can provide the directional functional interactions (i.e., effective connectivity) between the different brain regions underlying own- vs. other-race face recognition (Valdés-Sosa et al., 2006; Friston, 2011; Seth et al., 2015). Thus, the GCA is specifically suited to address such important questions as how the information from visual cortex transfer to senior cognitive cortex located in frontal lobe, or whether and how information transfer between two lateral hemispheres. This method of effective connectivity analysis has been widely used in EEG and fMRI time series analysis (Kamiński et al., 2001; Valdés-Sosa et al., 2006; for a review, see Seth et al., 2015). It also has been recently used in fNIRS data processing (Holper et al., 2012; Yuan, 2013).

Most of previous behavioral studies of developmental ORE focused their investigation on the children with age 3–13 years (e.g., Chance et al., 1982; Pezdek et al., 2003; Sangrigoli and de Schonen, 2004; Goodman et al., 2007; Anzures et al., 2014; Suhrke et al., 2014). For example, Chance et al. (1982) examined ORE in children from 6 to 14 years old, and found only older children (from 9 years on) showed ORE. Goodman et al. (2007) tested 5- to 13-year-old children, and also found the older children (from 8 years on) showed ORE. However, with improved methodologies, Anzures et al. (2014) found 5-year-old children had already showed ORE. Suhrke et al. (2014) even found significant ORE in 3-year-old children. These researches demonstrated that the age range of 3 to 13 is the crucial period for the development of ORE. In addition, this range of age is also the age period during which children's general face processing ability is thought to undergo a protracted development from immaturity in early childhood to maturity in late adolescence (Mondloch et al., 2003; for a review, see Lee et al., 2013). To uncover the neurodevelopmental pattern of face processing ability in general and that of the ORE in particular, the present study thus focused on children across this large age span.

We used the fNIRS methodology to obtain cortical neural response data in children between 3 and 13 years of age. Similar to fMRI, the fNIRS indirectly examines the brain activity through the measurement of the hemodynamic response (for reviews, see: Ferrari and Quaresima, 2012; Scholkmann et al., 2014). Although its spatial resolution is relatively inferior to fMRI, fNIRS presents some distinctive advantages for functionally neuroimaging the child population (Lloyd-Fox et al., 2010; Ding et al., 2014). First, fNIRS is much less susceptible to movement artifacts than fMRI and ERP, and is thus highly suitable for young children who are known to have difficult to control their spontaneous movements (Lloyd-Fox et al., 2010; Scholkmann et al., 2014). Second, fNIRS offers a less noisy and more comfortable experimental environment than fMRI to collect data from children in a more naturalistic manner (Scholkmann et al., 2014). Third, the operating costs of a NIRS machine are very limited, making it possible to test a large sample of children. Fourth, fNIRS signals are strongly correlated with those obtained with fMRI (Cui et al., 2011; Eggebrecht et al., 2012; Sato et al., 2013), making it possible to compare the results of fNIRS study with previous fMRI findings directly. Further and more relevant to the present research, compared to fMRI, fNIRS has higher temporal resolution (10 Hz) than the conventional fMRI (~1 Hz). Thus, fNIRS can more accurately characterize the temporal properties of the hemodynamic changes, making it specifically suitable for brain connectivity analysis (Scholkmann et al., 2014; Tak and Ye, 2014).

According to the recent functional neuroimaging findings regarding the development of face processing in children in general (Gathers et al., 2004; Aylward et al., 2005; Passarotti et al., 2007; Scherf et al., 2007) and functional connectivity specifically (Kadosh et al., 2011; Haist et al., 2013), regardless of face race, we expected more inter-area connections between the frontal and occipital brain areas with increased age, since previous research has found that the extended face network is hyper-activated around 10 years of age (Haist et al., 2013). Furthermore, according to the recent functional neuroimaging findings regarding the neural ORE in adults and children (Feng et al., 2011; Ding et al., 2014; Liu et al., 2015), we hypothesized that there would be differences in functional causal neural networks between own-race faces and other-race faces in children, and further this difference would change as a function of age. More specifically, there would be more inter-regional, but not intra-regional, connections (i.e., between the frontal and occipital brain areas) for own-race faces than other-race faces. In addition, according to the previous studies, because children have asymmetrical exposure to own-race vs. other-race faces (more exposure to the former than the latter), they would be more inclined to extract the identity level information from the visual input to process own-race faces than other-race faces. Thus, we hypothesized that with increased age there would be more connections from the occipital areas to the frontal areas for own-race faces than other-race faces.

## Materials and methods

### Participants

A total of 124 children (Male: 61; Mean age = 7.77 years, *SD* = 2.80 years, rang from 3.25 to 13.49 years) participated in the study. They were from preschools or primary schools in a medium sized city in Zhejiang Province, China, where 99.9% of the population was Han Chinese. All children were healthy, right-hand Han Chinese children with normal or correct-to-normal vision. According to the parental reports, they had never had direct contact with individuals from racial groups other than Han Chinese. A written informed consent was signed by parents or legal guardians of all children before their participation. Children also gave their oral consent. The present study was approved by the Ethics Committee of Zhejiang Normal University.

### Stimuli

The materials used in the present study included 40 black-and-white face photos of 20 female Chinese and 20 female Caucasian young adults. All photos were digitized in 24-bit depths with a resolution of 450 × 600 pixels and had not been seen by participants before the present study. Using Adobe Photoshop, we overlaid a black oval frame on the photo such that each face's ears, hair, and contour were concealed so as to prevent participants from using non-key face information to recognize the face. Each face was centered with a frontal view and presented in a neutral expression. In addition, the low-level physical attributes such as brightness and contrast were balanced using the SHINE program (Willenbockel et al., 2010).

### Experimental procedure

**Figure 1:**
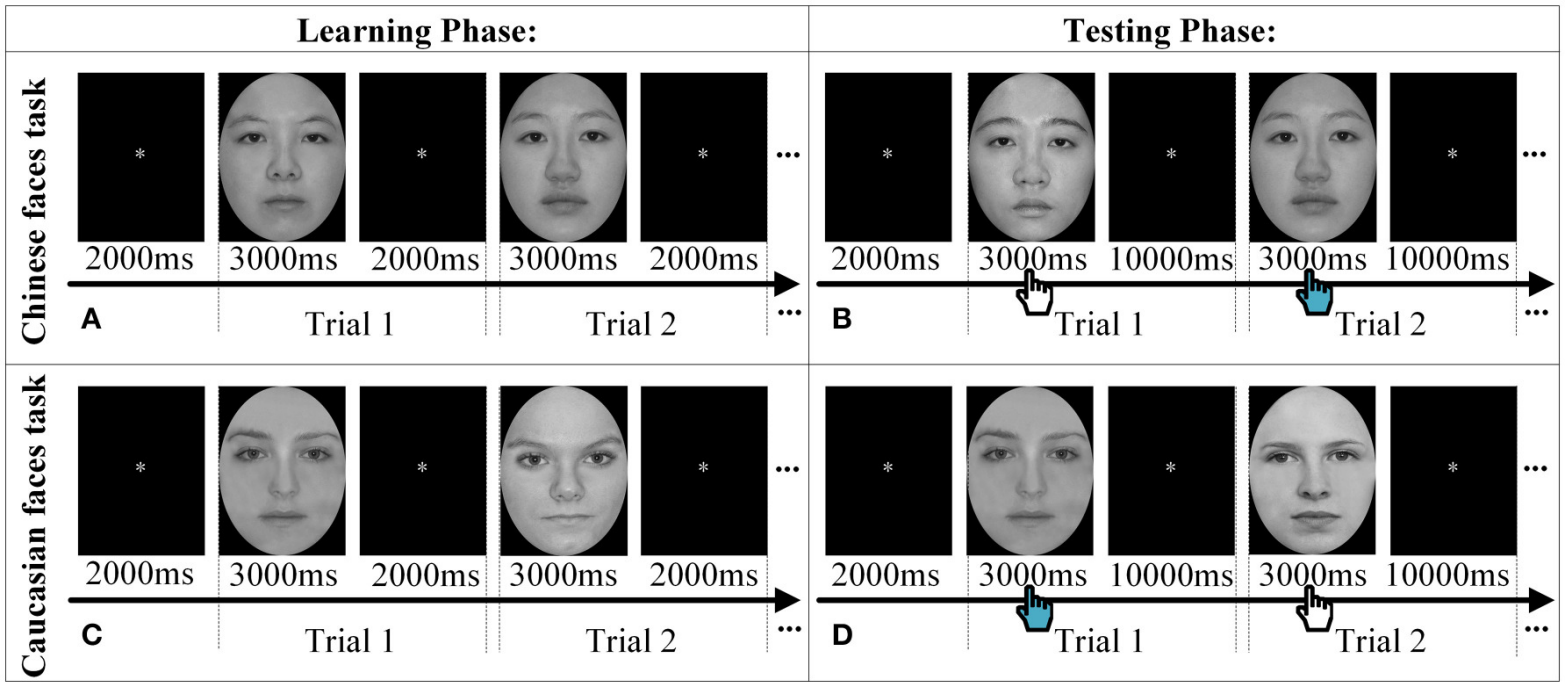
**The experimental procedure**. Two trials are displayed as samples to explain the procedure of Chinese faces task **(A,B)** and Caucasian faces task **(C,D)** in both the learning phase and the testing phase, respectively. The hand icon during the testing phase denoted that participants were required to make decision whether the face had been seen in the learning phase by pressing the keyboard.

We used an old/new paradigm to assess children's face recognition ability (Goodman et al., 2007; Ding et al., 2014). All of children were asked to perform two tasks, namely Chinese faces task (CH) and Caucasian faces task (CA), separately. The CH task only included Chinese face images and consisted of a learning phase followed by a testing phase. During the learning phase, each participant was required to remember 10 target Chinese faces successively (i.e., 10 trials in total), each of which was presented on the computer screen for 3 s followed by an interval of 2 s fixation (“*”). Then, during testing phase, the 10 “old” faces that had been learned were mixed with additional 10 “new” faces that had never been seen before. Each face image was presented for 3 s followed by an interval of 10 s fixation (“*”), during which the child was asked to make decision as to whether the currently seen face had been viewed in the learning phase via button presses. The brain functional data were recorded only in the testing phase because our research aimed to reveal neural activations associated with recognizing own- vs. other-race faces to reveal the neural ORE. The CA task used the same paradigm as that of CH task except that Chinese faces were replaced by Caucasian faces (see Figure 1). The order of two tasks was counterbalanced between participants.

Before the formal experiment, children were instructed to complete practice trials to insure that they understood the task requirements. The practice trials were identical to the experimental trials except that only four Chinese faces and four Caucasian faces were used. The faces were not used in the formal experiment.

### Behavior data acquisition

A Windows desktop computer presented the stimuli using E-prime 1.2 (PST Inc., U.S.A) and recorded children's behavior data. Elementary school children responded by pressing either “1” (an “old” target face) or “2” (a “new” foil face) themselves on the keyboard in each trial during the testing phase (key assignments were also counterbalanced between participants). However, for preschool children, considering their generally limited executive function ability, the experimenter pressed the keys on their behalf based on the children's oral report. Thus, no response latency data were directly collected from the preschool children. As a result, the present study only used recognition accuracy to measure both preschool and elementary school children's behavioral performance in own- and other-race face recognition.

### fNIRS data acquisition

**Figure 2:**
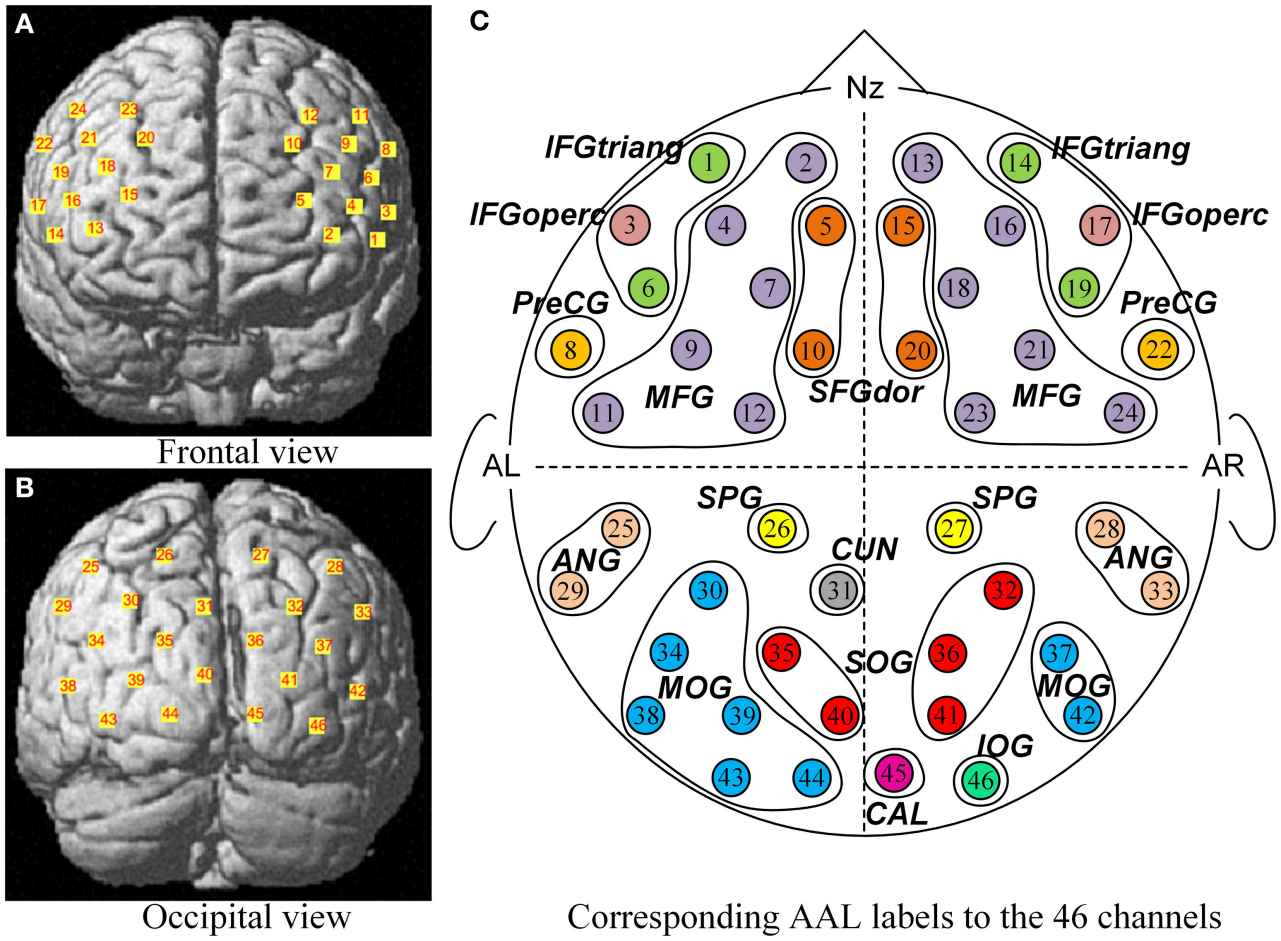
**The corresponding MNI locations and AAL labels of all channels**. The MNI locations of all channels were showed in the frontal view **(A)** and occipital view **(B)** respectively. No. 1 ~ No. 24 channels **(A)** were located almost entirely in the frontal lobe, and No. 25 ~ No. 46 channels **(B)** mainly covered the occipital cortex. From a sketch **(C)**, the 46 channels overlapped the 21 corresponding anatomical cortex regions according to the AAL template, namely SFGdor, the dorsolateral superior frontal gyrus; MFG, the middle frontal gyrus; IFGtriang, the triangular inferior frontal gyrus; IFGoperc, the opercula inferior frontal gyrus; PreCG, the precentral gyrus; SPG, the superior parietal gyrus; ANG, the angular gyrus; CUN, the cuneus; SOG, the superior occipital gyrus; MOG, the middle occipital gyrus; IOG, the inferior occipital gyrus; CAL, the calcarine cortex. L indicates the left hemisphere, and R indicates the right hemisphere.

A multichannel continuous-wave NIRS system (ETG-4000, Hitachi Medical Co., Japan) was used to acquire and record the optical density changes, from which we obtained the local changes of oxygenated hemoglobin ([oxy-Hb]) and deoxygenated hemoglobin ([doxy-Hb]) concentrations using the modified Beer-Lambert Law (Cope and Delpy, 1988; Scholkmann et al., 2014). ETG-4000 measures the optical density changes with two near-infrared lights (wavelengths ~695 and ~830 nm at each source fiber). Two sets of probes of ETG-4000 were mounted on a plastic helmet with a 30 cm source–detector distance, resulting in 46 measurement channels (See Figure 2). The placement of optodes was performed according to the international 10–20 system (Jasper, 1958). One set consisted of a pair of 3 × 3 arrays with five light emitters and four detectors, forming 24 channels, with its lowest probes positioned along the Fp1–Fp2 line to primarily cover the frontal cortex. The second other set consisted of a 3 × 5 array with eight light emitters and seven detectors, forming the 22 channels, with its lowest probes positioned along the O1–O2 line to primarily cover the occipital cortex. These measured brain areas are thought to be involved in processing faces stimuli (Haxby et al., 2000; Fairhall and Ishai, 2007; Ito and Bartholow, 2009). The sampling rate of ETG-4000 was set to 10 Hz. The communication between the task computer and ETG-4000 was implemented by E-prime.

After children finished the experiment, their relative spatial coordinates of the probes were measured by a 3D digitizer (PATRIOT, Polhemus Co., U.S.A), with its reference points defined as the following five anatomical landmarks: nasion, inion, Cz, the anterior tragus point of the left ear, and the anterior tragus point of the right ear. The coordinates of the probes were then used to calculate the coordinates of the 46 NIRS channels. These spatial data were then normalized using NIRS-SPM (Near Infrared Spectroscopy-Statistical Parametric Mapping) software package (Ye et al., 2009; Tak and Ye, 2014). We conducted spatial registrations of the NIRS channels according to the Montreal Neurological Institute (MNI) standard coordinate system using algorithms by Singh et al. (2005) (See Table 1). Then, the neuroanatomical labels for each channel locations were determined according to the widely adopted AAL (automated anatomical labeling) template (Tzourio-Mazoyer et al., 2002; See Figure 2). Additional, we obtained probabilistic estimations of the Brodmann area (BA) that each channel was on (Brodmann, 2007; See Table 1).

**Table 1:** **The corresponding MNI coordinate, AAL and Brodmann area for every NIRS channel**.

**MNI coordinates channel no. (*x*, *y*, *z*)**	**BA**	**AAL**	**MNI coordinates channel no. (*x*, *y*, *z*)**	**BA**	**AAL**
ch1 (−54, 36, 3)	45	L IFGtriang	ch13 (42, 59, 8)	46	R MFG
ch2 (−40, 59, 6)	10	L MFG	ch14 (57, 35, 5)	45	R IFGtriang
ch3 (−60, 16, 14)	44	L IFGoperc	ch15 (31, 63, 20)	10	R SFGdor
ch4 (−48, 45, 16)	45	L MFG	ch16 (50, 45, 18)	45	R MFG
ch5 (−29, 63, 18)	10	L SFGdor	ch17 (64, 14, 16)	6	R IFGoperc
ch6 (−54, 27, 26)	45	L IFGtriang	ch18 (39, 51, 29)	46	R MFG
ch7 (−37, 50, 27)	46	L MFG	ch19 (56, 27, 28)	45	R IFGtriang
ch8 (−59, 5, 35)	6	L PreCG	ch20 (25, 52, 39)	9	R SFGdor
ch9 (−45, 33, 37)	45	L MFG	ch21 (45, 34, 40)	9	R MFG
ch10 (−24, 52, 38)	9	L SFGdor	ch22 (61, 5, 38)	6	R PreCG
ch11 (−49, 14, 48)	9	L MFG	ch23 (31, 37, 49)	9	R MFG
ch12 (−32, 36, 47)	9	L MFG	ch24 (49, 14, 50)	9	R MFG
ch25 (−42, −69, 51)	39	L ANG	ch27 (16, −80, 54)	7	R SPG
ch26 (−18, −80, 55)	7	L SPG	ch28 (41, −71, 51)	7	R ANG
ch29 (−53, −69, 38)	39	L ANG	ch32 (27, −88, 38)	19	R SOG
ch30 (−31, −86, 40)	19	L MOG	ch33 (52, −71, 36)	39	R ANG
ch31 (−3, −89, 38)	19	L CUN	ch36 (14, −98, 25)	18	R SOG
ch34 (−42, −87, 26)	19	L MOG	ch37 (38, −89, 24)	19	R MOG
ch35 (−17, −98, 26)	18	L SOG	ch41 (25, −101, 11)	17	R SOG
ch38 (−51, −81, 11)	19	L MOG	ch42 (48, −84, 8)	19	R MOG
ch39 (−28, −99, 12)	17	L MOG	ch45 (15, −105, 0)	17	R CAL
ch40 (−4, −104, 13)	17	L SOG	ch46 (35, −96, −4)	18	R IOG
ch43 (−38, −94, −2)	18	L MOG			
ch44 (−16, −106, 0)	17	L MOG			

*IFG indicates the inferior frontal gyrus, SFG indicates the superior frontal gyrus, MFG indicates the middle frontal gyrus, SOG indicates the superior occipital gyrus, MOG indicates the middle occipital gyrus, PG indicates the post-central gyrus, AG indicates the angular gyrus, and L means left hemisphere, while R means right hemisphere*.

### Preprocessing for fNIRS data

Although we obtained the [oxy-Hb] and [doxy-Hb] concentration change data concurrently, for the present study, we only analyzed the [oxy-Hb] data because [deoxy-Hb] signals have been found to be highly noisy and unreliable (Strangman et al., 2002; Homae et al., 2007). The fNIRS data preprocessing was implemented in NIRS-SPM software package based on MATLAB. The time series of the [oxy-Hb] concentration changes for each channel was first low-pass filtered with the shape of the canonical hemodynamic response function (HRF) in statistical parametric mapping (SPM; Friston et al., 2003; Ye et al., 2009). Then, the detrending based on the wavelet-minimum description length algorithm (Wavelet-MDL) was applied to eliminate global drift produced by breathing, cardiac motion, vasomotion, body motion, and so on (Jang et al., 2009; Ye et al., 2009). In other words, the preprocessing of fNIRS data removed low- and high-frequency noises to improve the signal-to-noise ratio so as to extract the task related fNIRS signals. In addition, the baseline correction by linear least-squares fitting was performed for each channel based on the two baseline data: the mean of 2-s periods pre-/post-task sections.

### Granger causality analysis (GCA)

To investigate whether and how own- and other-race faces recognition processes differed in terms of functional neural connectivity, we performed the Granger causality analysis (GCA) with the preprocessed [oxy-Hb] time series data. GCA is able to explore the directed effective connectivity among multiple brain cortices based on vector auto-regressive models (VAR; Seth et al., 2015), and thus offers the potential for defining the functional circuits underlying neural interactions (Goebel et al., 2003; Yuan, 2013). Concerning the two face race tasks in the present study, we tested Granger causality for every participant under either the own- or other-race faces task conditions, respectively. More specifically, a Granger causality testing was accomplished in the following steps:

First, a linear auto-regression model of each channel's preprocessed [oxy-Hb] temporal sequence data based on their past observations was built to estimate their error of prediction. For example, assuming that *X*_*A*_ and *X*_*B*_ were time series of Channel *A* and Channel *B*, respectively. *X*_*A*_ and *X*_*B*_ could be fitted with two autoregressive models of order p (Goebel et al., 2003):

$$\begin{array}{l} X_{A}\left(t\right)=a_{0}+\overset{p}{\underset{\left(i=1\right)}{\sum }}a_{i}X_{A}\left(t-i\right)+\varepsilon_{A}\left(t\right) \end{array}$$

$$\begin{array}{l} X_{B}\left(t\right)=b_{0}+\overset{p}{\underset{\left(i=1\right)}{\sum }}b_{i}X_{B}\left(t-i\right)+\varepsilon_{B}\left(t\right) \end{array}$$

Where *p* determined by Bayesian information criterion (BIC), and the residuals of the two models (i.e., ε_*A*_ and ε_*B*_) were the white noise process (Yuan, 2013).

Next, the auto-regression of *X*_*A*_ was augmented by including previous observations of *X*_*B*_; similarly, the auto-regression of *X*_*B*_ was augmented by including previous observations of *X*_*A*_:

$$\begin{array}{l} X_{A}\left(t\right)=a_{0}^{\prime }+\overset{p}{\underset{\left(i=1\right)}{\sum }}a_{i}^{\prime }\;X_{A}\left(t-i\right)+\overset{p}{\underset{\left(i=1\right)}{\sum }}b_{i}^{\prime }X_{B}\left(t-i\right)+\varepsilon_{BA}\left(t\right)\\ X_{B}\left(t\right)=b_{0}^{\prime }+\overset{p}{\underset{\left(i=1\right)}{\sum }}b_{i}^{\prime }\;X_{B}\left(t-i\right)+\overset{p}{\underset{\left(i=1\right)}{\sum }}a_{i}^{\prime }X_{A}\left(t-i\right)+\varepsilon_{AB}\left(t\right) \end{array}$$

Then, we would test whether the inclusion of past observations of one channel particularly benefited the reduction of another's prediction error to define the causal relationship between these two channels (Goebel et al., 2003; Sato et al., 2010; Yuan, 2013). We defined the magnitude of Granger causality from *B* to *A* as:

$$\begin{array}{l} G_{\left(B\to A\right)}=\ln\;\frac{COV\left(\varepsilon_{BA}\right)}{COV\left(\varepsilon_{A}\right)} \end{array}$$

In other words, according to Granger causality a variable *B* “causes” a variable *A* if the past of *B* helps predict the future of *A* better than can be done by knowing only the past of *A*. Similarly, we defined the magnitude of Granger causality from *A* to *B* as:

$$\begin{array}{l} G_{\left(A\to B\right)}=\ln\;\frac{COV\left(\varepsilon_{AB}\right)}{COV\left(\varepsilon_{B}\right)} \end{array}$$

In the present study, for each participant and under each task condition, the causal map was obtained by calculating the weights of Granger causal relations between the time series of [oxy-Hb] of each pair of channels. For each pair of channels, a two-way repeated-measure analysis of variance (ANOVA) was performed on their GCA weights with the face race task (own-race face vs. other-race face) as the within-subject factor and the age as the between-subject factor (continuous variable).

To ensure reliability of the ANOVA results and reduce the likelihood of false discoveries, capitalizing the large sample size of the present study, we used the Bootstrapping-based method. Bootstrapping is a method to verify the reliability of statistical findings with the use of random down-sampling of the sample of the data on hand. It has been widely used in various fields (Davison and Hinkley, 1997; Lee and Rodgers, 1998; Burleigh et al., 2006). Specifically, we first randomly down-sampled 50 percent of the participants to form a subset, then we performed the ANOVA of face race by age on the participants' GCA weights in this subset. Then, instead of performing an *F*-test, we only computed the partial eta-squared effect size statistics ($\eta_{\text{p}}^{2}$) in ANOVA. We repeated this step 10,000 times. As a result, we obtained 10,000 partial eta-squared effect size statistics. Finally, we obtained the averaged effect size by calculating the mean value of these 10,000 partial eta-squared effect size statistics.

Then, to estimate the statistical significance of the averaged effect size, we conducted a permutation test. First, we down-sample 50% of the participants as a new sample and randomly scrambled the participants' GCA weights from the own- race and other-race conditions into two new pseudo conditions (a pseudo “own-race” condition and a pseudo “other-race” condition). We then performed the same ANOVA as mentioned above to obtain the partial eta-squared effect size statistics of the scrambled data set. Again, we repeated this step 10,000 times to obtained 10,000 partial eta-squared effect size statistics that forms a distribution of the effect sizes based on the scrambled data. Using this random distribution of effect sizes, we then compared the mean effect size based on the real data to determine whether the mean effect size was significantly different from the chance. We considered the effect sizes to be significant only when they were in the top 5% of the random distribution.

## Results

Because all participants of the present study were Chinese, we will henceforth refer to Chinese faces as own-race faces while Caucasian faces as other-race faces, respectively.

### Behavior results

According to previous studies, we referred to better recognition performance for own-race faces than own-race faces as ORE. The mean accuracy of own-race faces recognition and that of other-race faces was 66.01 ± 14.86% and 64 ± 13.97%, respectively. The repeated measure 2 (stimulus race: own vs. other race) × 1 (age in years: continuous) ANOVA was performed on accuracy with stimulus race as a within-subject variable and children's age as a continuous variable. Only the main effect of age was significant, *F*_(1, 122)_ = 88.72, *p* < 0.001, $\eta_{\text{p}}^{2}$ = 0.42: with increased age, children's accuracy improved significantly for both own- and other-race faces. Neither the interaction nor the main effect of face race was significant [The interaction effect: *F*_(1, 122)_ = 0.04, *p* = 0.84, $\eta_{\text{p}}^{2}$ < 0.001; the main effect of face race: *F*_(1, 122)_ = 0.60, *p* = 0.44, $\eta_{\text{p}}^{2}$ < 0.001; See Table 2].

**Table 2:** **Means (***SD***) recognition accuracy of Own- and Other-race faces and results of one repeated measure of ANOVA with face race as a within-subject variable and age as a continuous variable**.

**Mean age ±*SD* (year)**	**Mean accuracy (*SD*)**	**ANOVA**
7.77 ± 2.80 (3.25 ~ 13.49)	Own-race faces: 0.66 (0.15)	Age: *F*_(1, 122)_ = 88.72, *p* < 0.001, η*p*^2^ = 0.42;
		Face race: *F*_(1, 122)_ = 0.60, *p* = 0.44, η*p*^2^ < 0.001;
	Other-race faces: 0.64 (0.14)	Age × face race: *F*_(1, 122)_ = 00.04, *p* = 0.84, η*p*^2^ < 0.001

*SD indicates standard deviation*.

### GCA results

We obtained two Granger causal networks, one for own-race faces, and the other for other-race faces. To obtain own-race face Granger causal networks, the effective connectivities were calculated with different intensities and directions among the 46 channels while children were recognizing the own-race faces. To obtain the other-race face Granger causal networks, the effective connectivities were calculated with different intensities and directions among the 46 channels while children were recognizing the other-race faces. In keeping with the definition for the behavioral ORE for own-race faces relative to other-race faces, we defined the Neural ORE as the differences in effective connectivity intensity between own-race faces causal networks and other-race face causal networks.

To examine whether the own-race face causal networks differed from the other-race face causal networks in children, as mentioned above, we conducted 2 (race: own-race faces vs. other-race faces) × 1 (age in years: continuous) repeated measures ANOVAs with intensity of each effective connectivity as the dependent variable. Specifically, we used the Bootstrapping method mentioned above to perform significance test. Here we report the significant test results based on the Bootstrapping.

**Figure 3:**
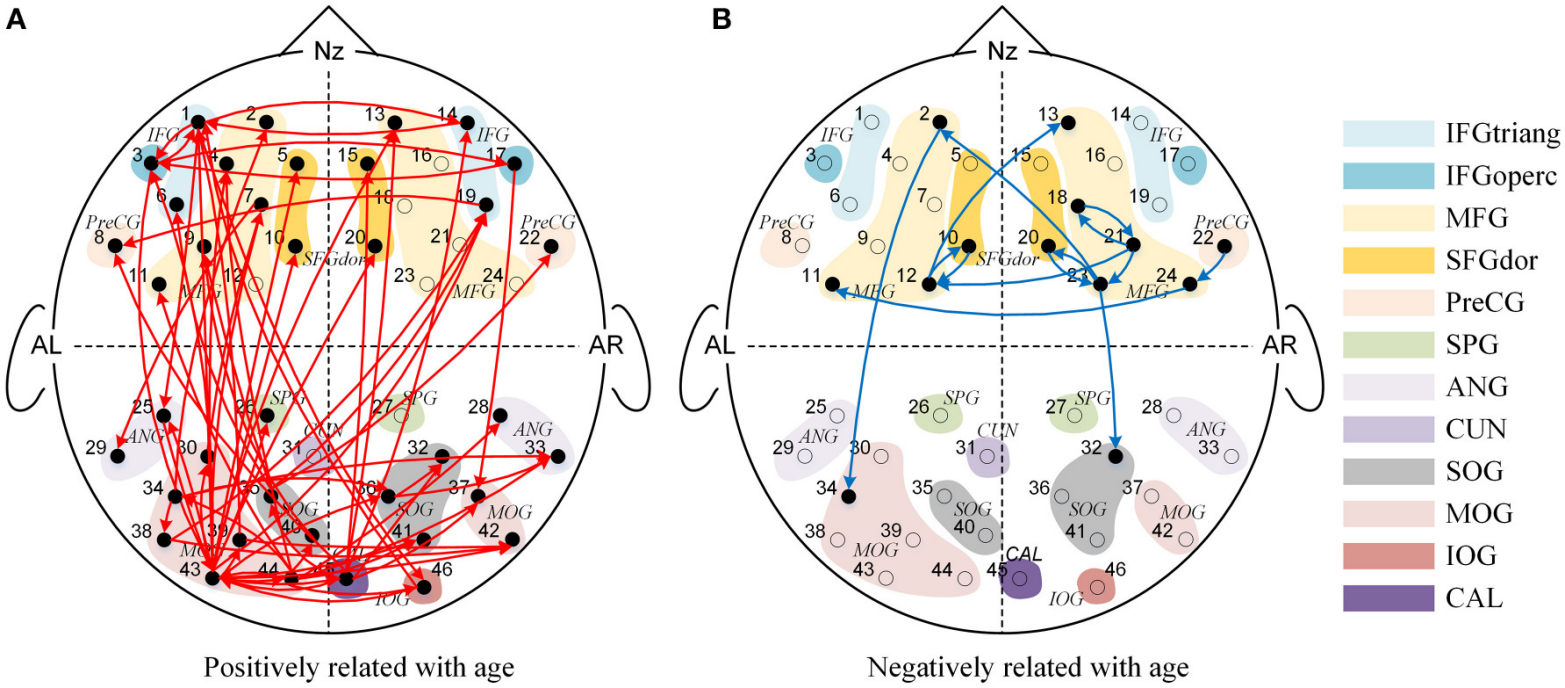
**The Granger causal network with a significant main effect of age**. The red connections in **(A)** presented the positive correlation between age and Granger weights regardless of face race, while the blue connections in **(B)** presented the negative correlation between age and Granger weights regardless of face race.

We found significant main effects of age for 77 effective connections (See Figure 3). Among them, 63 connections in Figure 3A increased significantly with age (*P'*s < 0.05), whereas 14 connections in Figure 3B decreased with increased age (*P'*s < 0.05). These causal connections revealed the overall developmental pattern in face processing from 3 to 13 years of age. More specifically, regardless of face race, more causal connections were strengthened with the increased age. Among them, 31 were inter-area connections (4 connections from the frontal area to the occipital area, 27 connections from the occipital area to the frontal area) and 32 were intra-area connections (25 connections in the occipital area, and 7 connections in the frontal area). In contrast, only a few connections weakened with increased age, among which 2 were inter-area connections (from the frontal area to the occipital area) and 12 were intra-area connections (all in the frontal area).

**Figure 4:**
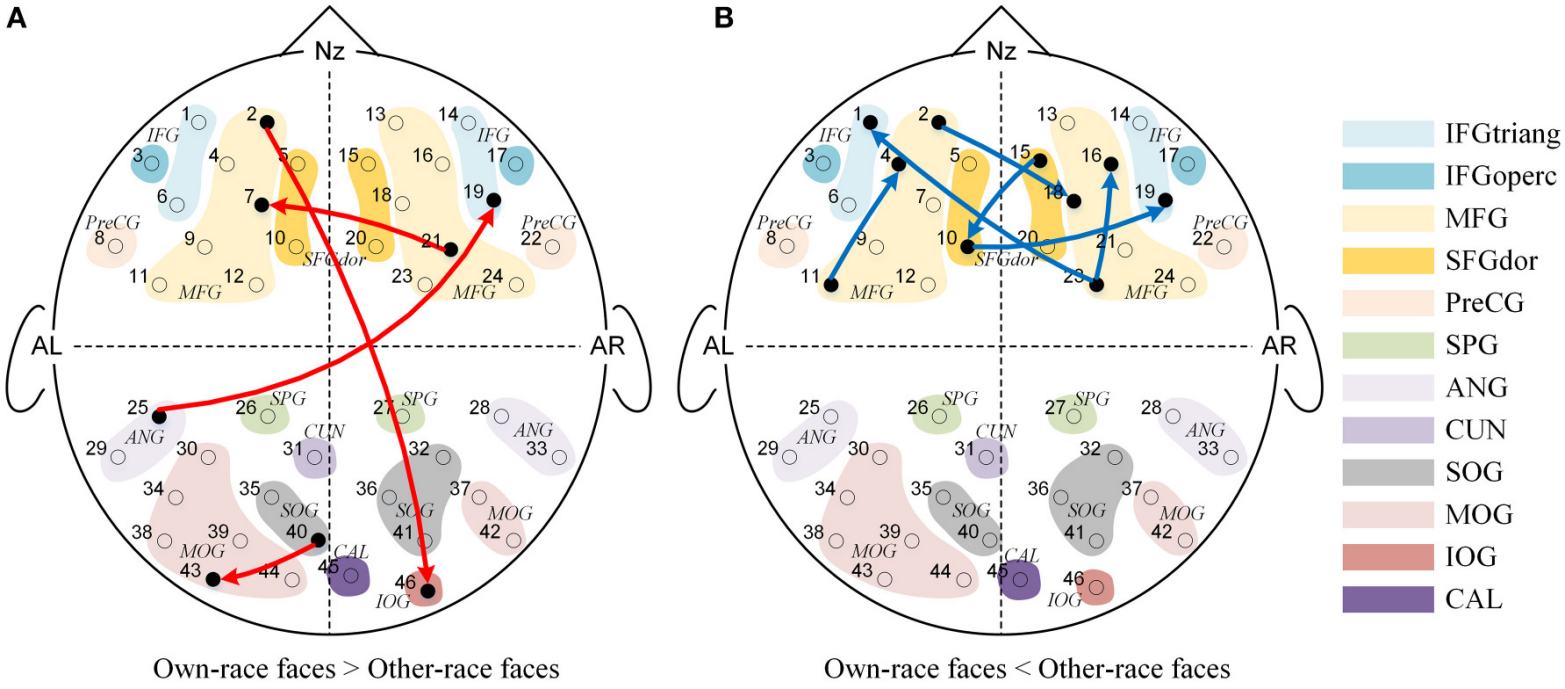
**The Granger causal network with a significant main effect of Face race**. The red connections in **(A)** showed that the own-race faces elicited greater Granger causality than that of other-race faces regardless of age, while the blue connections in **(B)** showed that the other-race faces elicited greater Granger causality than that of own-race faces regardless of age.

We also found significant main effects of face race for 10 causal connections (See Figure 4), suggesting that regardless of age, the effective connectivities differed significantly for own-race vs. other-race faces causal networks. More specifically, as shown in Figure 4A, 4 effective connections in the own-race face causal network were stronger than those in the other-race face network (*P'*s < 0.05). Among these connections, 2 were inter-area connections (1 connection from the frontal area to the occipital area, and 1 connection from the occipital area to the frontal area) and 2 were intra-area connections (1 connection in the frontal area, and 1 connection in the occipital area). In contrast, there were 6 connections from the other-race face causal network that were stronger than those in the own-race face network (*P'*s < 0.05; See Figure 4B). These connections were intra-area connections (all connections in the frontal area).

**Figure 5:**
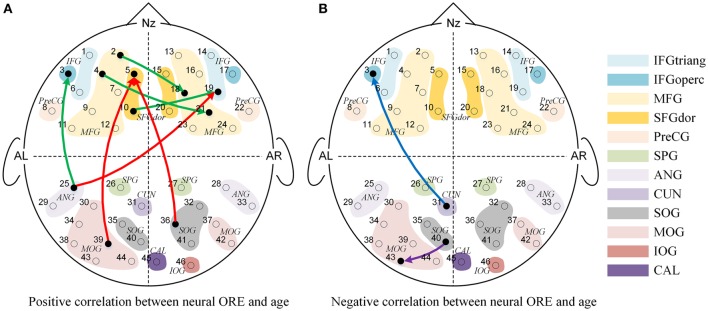
**The Granger causal network with a significant interaction effect of Face Race (Chinese faces task vs. Caucasian faces task) and Age (from 3 to 13 years-old). (A)** Presented a positive correlation between the Neural ORE and age, while **(B)** presented a negative correlation between the Neural ORE and age. The red and green connections in **(A)** represented two subtypes of positive development. Similarly, the blue and purple connections in **(B)** represented two subtypes of negative development.

**Figure 6:**
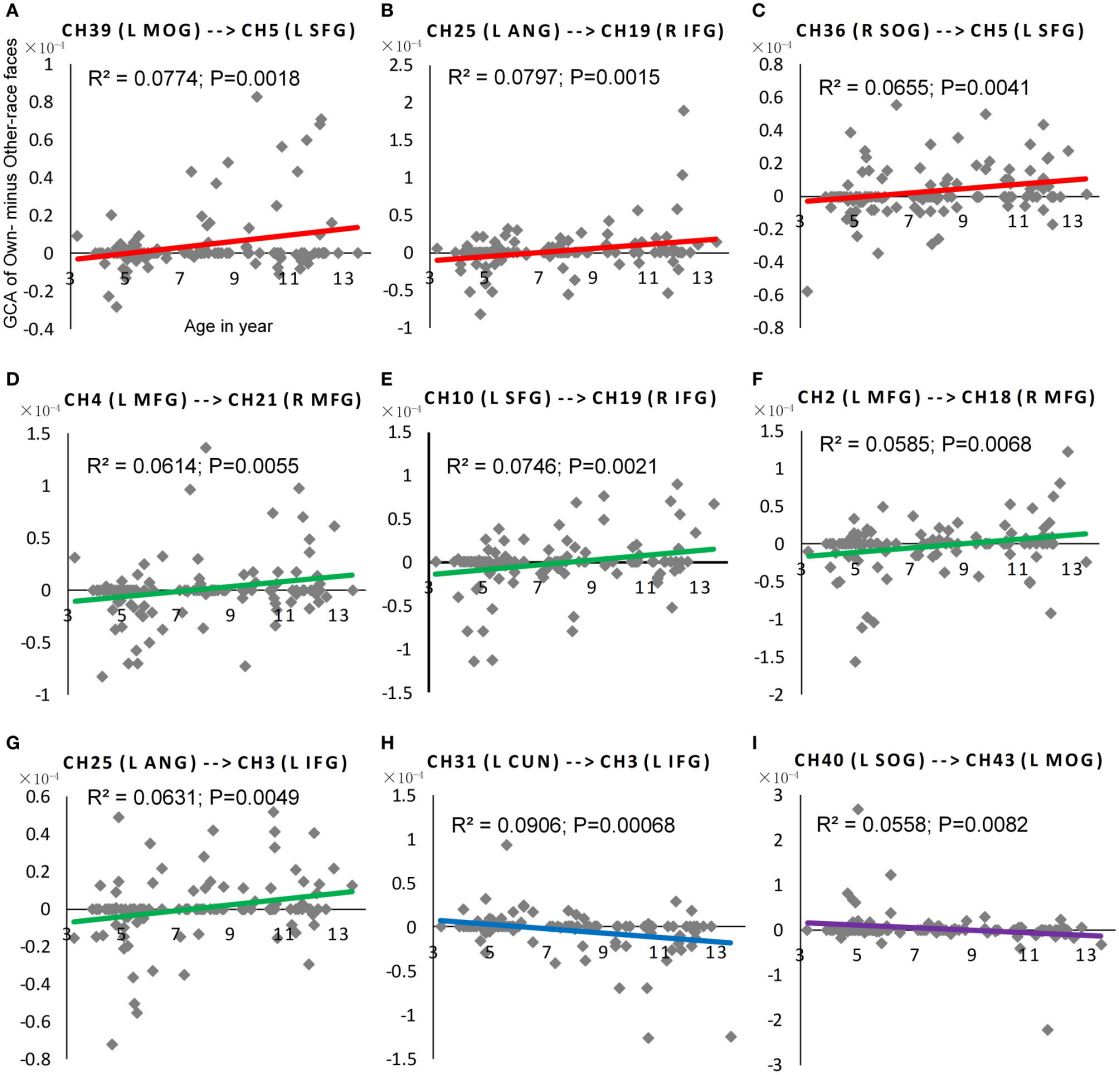
**The scatter plot of Pearson correlations between Age in years and the NORE**. The red plots in **(A–C)** were corresponding scatter plot of Pearson correlation for the first positive subtype, and the green plots in **(D–G)** were corresponding scatter plot of Pearson correlation for the second positive subtype. Similarly, the blue plot in **(H)** were corresponding scatter plot of Pearson correlation for the first negative subtype, and the purple plots in **(I)** were corresponding scatter plot of Pearson correlation for the second negative subtype.

Further and specifically relevant to our age-related hypotheses regarding the neural ORE, the repeated measures ANOVAs yielded significant face race by age interactions for nine connections (*p'*s < 0.05; See Figure 5). To future explore these significant interactions, we calculated the Pearson correlations between age in years and the Neural ORE. The results also are presented in Figure 6 and Table 3.

**Table 3:** **The coefficients of Pearson correlation between age in years and the NORE for the connections with the significant interactions**.

**From channel no**.	**To channel no**.	***r***	***p***	**From channel no**.	**To channel no**.	***r***	***p***
39	5	0.2782	0.0018	4	21	0.2477	0.0055
25	19	0.2824	0.0015	10	19	0.2731	0.0021
36	5	0.2560	0.0041	2	18	0.2419	0.0068
31	3	−0.3009	0.00068	25	3	0.2513	0.0049
40	43	−0.2363	0.0082				

As shown in Figure 5, nine connections with the significant interactions had two different trends. Seven connections (Figures 5A, 6A–G) presented a positive correlation between the Neural ORE and age: The neural ORE were significantly greater with increased age. There were two subtypes of developmental patterns associated with this positive correlation. One positive pattern was that the connection intensity of 3 inter-area connections (all from the occipital area to the frontal area) in the own-race face causal network increased with age but those in the other-race face causal network did not (Figures 6A–C, 5A red lines). The other pattern was that the intensity of 3 intra-area connections (all in the frontal area) and 1 inter-area connection (from the occipital area to the frontal area) in the own-race causal network increased with age whereas those in the other-race face network decreased with age (Figures 6D–G, 5A green lines).

In addition, there were the other two connections whereby the Neural ORE decreased with age (Figures 5B, 6H,I). Among them, there were also two subtypes in developmental patterns associated with this negative correlation. One pattern was that the intensity of one inter-area connection (from the occipital area to the frontal area) in the other-race face network increased with age but that in the own-race face network did not (Figures 6H, 5B blue line). The other pattern was that the intensity of one intra-are connection (in the occipital area) in the other-race network increased with age, whereas that in the own-race face network decreased with age (Figures 6I, 5B purple line). For the cortical areas that formed significant causal connections described above, see Table 4.

**Table 4:** **The results of interaction and the 2 student's ***t***-test for them**.

**Connection from Ch no. to Ch no**.	**Interaction effect**			***Post-hoc***							
	**F statistics**	**Sig**.	**Partial η^2^**	**Pair-*t*-Test (T statistics; Sig.) CH minus CA**				**Two sample-*t*-Test (T statistics; Sig.) younger minus older**			
				**Younger group**		**Older group**		**Chinese faces**		**Caucasian faces**	
2 → 18	7.584	0.0068^**^	0.0585	−2.267	0.0269^*^	0.94	0.351	−2.486	0.0143^*^	1.236	0.2187
4 → 21	7.975	0.0056^**^	0.0614	−1.949	0.0559	1.865	0.067	−1.862	0.065	1.828	0.07
10 → 19	9.836	0.0021^**^	0.0746	−2.247	0.0283^*^	1.552	0.1257	−2.443	0.0160^*^	1.517	0.1318
25 → 3	8.223	0.0049^**^	0.0632	−1.987	0.0514	2.821	0.0064^**^	−2.183	0.0310^*^	2.42	0.0170^*^
25 → 19	10.572	0.0015^**^	0.0798	−1.485	0.1428	1.737	0.0875	−2.382	0.0188^*^	0.452	0.6517
31 → 3	12.15	0.0006^***^	0.0906	1.163	0.2495	−2.78	0.0072^**^	1.633	0.105	−2.575	0.0112^*^
39 → 5	10.231	0.0018^**^	0.0774	0.01	0.9921	3.353	0.0014^**^	−3.045	0.0029^**^	0.499	0.6185
40 → 43	7.216	0.0082^**^	0.0558	2.082	0.0416^*^	−1.24	0.2199	1.782	0.0773	−1.461	0.1466
36 → 5	8.553	0.0041^**^	0.0655	−0.102	0.9194	3.052	0.0034^**^	−1.862	0.065	1.258	0.2109

*The 2 student's t-test based on two divided groups, namely younger and older group. One was the Pair-T-test for younger group and older group, respectively, between own-race faces and other-race faces. The other was the Two-sample T-test for own-race and other-race, respectively, between younger group and older group*.

* Sig. < 0.05;

** Sig. < 0.01;

*** *Sig. < 0.001*.

## Discussion

The present study, using fNIRS methodology, examined the Neural ORE by comparing the causal network differences between own-race face and other-race face recognition in a large sample of preschool and elementary school children. We first obtained the Granger causal connectivity between each pair of channels when children with different ages recognized own-race faces and other-race faces, and then performed repeated measures ANOVAs on each Granger causal connectivity with age and face race (own-race faces vs. other-race faces) as independent variables. To ensure result reliability and minimize false discoveries, we used the Bootstrapping-based method. To our knowledge, this is the first study to explore the developmental trajectory of the neural ORE on the basis of effective connectivity analyses.

### About the granger analysis results

First, consistent with our hypothesis, we found robust and widespread significant main effects of age. This effect revealed that, regardless of face race, the overall causal connectivity underlying individual face recognition changed with age. We found that the majority of causal connections increased with age, whereas only a few of connections decreased with age. These findings suggest that with increased age, children recruit an increasingly more complex network for face recognition. This age-related change in functional connectivity parallels that in behavior. It is well-established that as age increases, children develop greater expertise in face recognition. More specifically, they become increasingly able to gather larger amounts of information about the faces they see and do so at faster speeds (Mondloch et al., 2003, 2010; Lee et al., 2013). Our findings suggest that the increase in the ability to recognize faces efficiently may involve an increase in functional connectivity complexity between the different brain regions of the extended face processing network. This increase in network functional connectivity may be needed to support the different stages of face processing, such as perception of face shape, integration of local face features, and retrieval of personal information, and face sematic information (e.g., race, gender, familiarity).

We found that the age-related enhancement of functional connectivity included two patterns of connections: One was the increase in the intra-area connection inside the occipital visual cortex or inside the frontal cortex, and the other was in the inter-area connection between the occipital visual cortex and the frontal cortex. Regarding with the intra-area connection, the occipital visual cortex is believed to be part of the core face network and important for face feature detection (Pitcher et al., 2011; Arcurio et al., 2012; Joseph et al., 2012). Previous studies also found that the OFA/FFA connectivity in the occipital area increased with age (He et al., 2015; Song et al., 2015). Thus, although fNIRS is unable to reach the depth of OFA/FFA, the increased connectivity inside the occipital area indicated that children become more sensitive to the face features with increased age. The frontal cortex areas, especially the MFG and IFG, are believed to be involved in more complex face processing such as linking faces to sematic information such as familiarities, categories (e.g., race; Haxby et al., 1996, 2000; Ishai et al., 2005; Fairhall and Ishai, 2007). Thus, the increased connectivity inside the frontal cortex suggested that children became increasing active in integrating face sematic information. Regarding with the inter-area connection, since the face processing involves a core network in the occipital-temporal cortex (Rossion et al., 2003; Pitcher et al., 2011) and the extended network that includes the frontal cortex (Renzi et al., 2013), it is not surprising that, with increased age, children had increased connectivity between the occipital cortex and the frontal cortex. In addition, we also found that with increased age, there were more effective connectivity from the occipital area to the frontal area than from the frontal area to the occipital area. This finding is consistent with the finding of Joseph et al. (2012) who found increased connectivities between the primary visual area and the right inferior orbitofrontal area as the age increased. It was suggested that this increase reflected an increased face processing expertise to process faces at the individual level.

Second, we also found that only a limited number of connections showed significant main effects of face race on the functional connectivity between different cortical areas, in sharp contrast to the large number of connections showing the main effect of age. This is consistent with the behavioral and neuroimaging findings that although both behavioral and neural OREs are often observed in children and adults (Chance et al., 1982; Walker and Hewstone, 2006; Scott et al., 2007; Anzures et al., 2014; Ding et al., 2014; Liu et al., 2015), the size of the main effects of face race is typically small. The present finding is consistent with this general finding, suggesting that there may exist a limited number of functional connections that differentiate between own- and other-race faces regardless of age. One possibility is that the differentiation between own- and other-race faces may change with increased age.

Third, we found significant face race by age interactions for nine connections, indicating that the differences in effective connectivity elicited by own-race faces relative to other-race faces were indeed modulated by age. For the connectivities with significant interaction effects, some of them showed a positive correlation between children's age and the difference in causal connectivity of own-race faces relative to other-race faces. In other words, the older the children's age, the greater the causal connectivity elicited by own-race faces than other-race faces. In fact, this positive correlation between age and ORE-related connectivity has two developmental patterns.

The first pattern was that there were initially no significant differences in connectivity between own-race face and other-race faces in younger children. However, with increased age, the causal connectivity elicited by own-race faces was increasingly enhanced, whereas those elicited by other-race faces did not change with age. Thus, in the end, with increased age, own-race faces elicited stronger causal connectivity than that elicited by other-race faces. This developmental pattern was seen among connections from the occipital visual cortex to the frontal cortex (i.e., the right IFG and the left SFG, Figure 5A, red lines). It has been suggested that the right IFG is part of the extended neural system for face recognition, and this region plays a crucial role in the storage and retrieval of such semantic information as familiarity, race, and identity (Haxby et al., 1996, 2000; Fairhall and Ishai, 2007; Ito and Bartholow, 2009). The left SFG is also reported to be involved in memory processing of faces (Petrides, 2005; Li et al., 2013). With increased age, there is an increased demand for children to learn to discriminate individuals due to their expanding social milieu that typically only involve own-race individuals. As a result, children's core and extended face processing network might need to be coupled more closely to address this increased processing demand, which might become automated with increased experience in processing own-race faces (Levin, 2000; Gauthier and Nelson, 2001; Lee et al., 2013; Senholzi and Ito, 2013; Liu et al., 2015).

In contrast, people have few experience of other-race face recognition, and therefore fail to automatically recognize other-race faces at individual level (Levin, 2000; Young et al., 2012; Lee et al., 2013; Liu et al., 2015). Although in the present study, both own-race faces and other-race faces are asked to be individually recognized, the mechanism of the individual processing of other-race faces is different from that of automatic individual processing of own-race faces (Levin, 2000; Senholzi and Ito, 2013; Liu et al., 2015). As mentioned before, the own-race faces need to extract more information from visual input or take finer processing on this visual information, and accordingly was represented by the stronger interaction or connectivity from the occipital visual cortex to the frontal cortex.

The second positive pattern was that the other-race faces initially elicited significantly stronger causal connectivity than own-race faces in younger children. However, with increased age, the causal connectivity elicited by own-race faces was enhanced, whereas those elicited by other-race faces either did not change with age or even decreased. Thus, in the end, own-race faces elicited connectivity either equal to or even stronger causal connectivity than, that elicited by other-race faces. This developmental pattern was mainly seen among connection from the left frontal cortex to the right frontal cortex (Figure 5A, green lines). Recent studies have suggested that the left hemisphere may play an important role in the processing of local features (e.g., facial feature), whereas the right hemisphere may play a dominant role in holistic processing (Hillger and Koenig, 1991; Maurer et al., 2007; Cattaneo et al., 2014). The automatic individual recognition of own-race faces is highly dependent on holistic processing (Rhodes et al., 2006; Michel et al., 2006a,b, 2010; Hancock and Rhodes, 2008). With increased age, the gradually increased expertise at processing own-race faces might have led to more automatic holistic processing that needs to integrate both local feature information (e.g., the shapes of eyes) and spatial information (e.g., the relative spatial relations between facial features). Thus, it is likely that this positive pattern reflected age-related changes in connectivity from the left frontal cortex to the right cortex for automatic integration of local information into holistic processing.

We also found two negative age-related patterns: As age increased, causal connectivity elicited by other-race face became greater than own-race faces. One of them was the causal connectivity from the left cuneus to the left IFG (Figure 5B, blue lines). For this connection, there are no significant differences of causal connectivity between own-race faces and other-race faces among the younger children. However, this connection presented stronger connectivity for other-race faces than own-race faces among the older children. The enhanced connectivity of this connection when comparing other-race faces to own-race faces may be due to an additional demand to decode other-race face feature information (Levin, 2000; Senholzi and Ito, 2013; Liu et al., 2015). Another causal connectivity with negative correlation was from the left SOG to the left MOG (Figure 5B, purple lines). This connection shows stronger for own-race faces than other-race faces at the younger age, but with increased age, the difference between own- and other-race disappeared. This finding was unexpected and awaits further verification and investigation.

### About the behavior results

It should be noted that the present study failed to obtain behavioral ORE from our child participants. Although the robust behavioral ORE has been well-established in adults, there is great inconsistence about the behavior ORE of children (Goodman et al., 2007; for a review, see Anzures et al., 2013b). As children are sensitive to the changes of experimental conditions, the inconsistence of the behavior ORE among the existing studies might be due to the different experimental environments or methods used in these studies. For example, to prevent children from using the non-key facial features (e.g., face contour or hairstyle) to recognize faces, we used an oval to cover such cues, which might have made the stimuli less naturalistic. Indeed, previous studies that used full faces typically produced behavioral ORE (e.g., Anzures et al., 2010), whereas those that used stimuli similar to ours failed to obtain behavioral ORE (e.g., Hu et al., 2014). Nevertheless, despite the lack of behavioral ORE, we found significant differences in neural network connectivity between own-race faces and other-race faces. Our neural imaging finding thus suggests that the neural responses might be more sensitive than behavior responses to own- vs. other-race face processing differences, in line with the suggestion of Balas et al. (2011).

## Conclusion

The present study examined the causal neural networks for own- and other-race face recognition in preschool and elementary school children. We found significant age-related increases in functional connectivity in children's face processing neural network in general and inter-regional connectivity specifically, paralleling a similar age-related improvement in behavioral face processing ability. In addition, we also found that significant differences in neural functional connectivity between the recognition of own-race faces and that of other-race faces were modulated by age. These finding suggested that like the behavioral ORE, the neural ORE also emerges in early childhood and undergoes a protracted developmental course. The present study also demonstrated the advantages of using fNIRS methodology to study the neural functional connectivity associated with perceptual-cognitive development.

## Author contributions

Experimental design and data recording: JL, GF, KL, and XD; Experimental data analyze: GZ and JL; Manuscript writing: GZ and JL; Manuscript revision: JL, KL, and XD.

### Conflict of interest statement

The authors declare that the research was conducted in the absence of any commercial or financial relationships that could be construed as a potential conflict of interest.
